# B Chromosome System in the Korean Field Mouse *Apodemus peninsulae* Thomas 1907 (Rodentia, Muridae)

**DOI:** 10.3390/genes9100472

**Published:** 2018-09-27

**Authors:** Yuri M. Borisov, Igor A. Zhigarev

**Affiliations:** 1 Severtzov Institute of Ecology and Evolution, Russia Academy of Sciences, Moscow 119071, Russia; boriss-spb@yandex.ru; 2 Institute of Biology and Chemistry, Moscow State University of Education (MSPU), Moscow 129164, Russia

**Keywords:** B chromosomes, dot-like (micro) Bs, karyotypic characteristics, Вs, B morphotypes, *Apodemus peninsulae*

## Abstract

In this paper, we analyzed B chromosome variation in Korean field mouse *Apodemus peninsulae* Thomas 1907 (Rodentia, Muridae) based on a 40-year study of karyotypes collected from geographically distant populations in East Siberia, North Mongolia, China, the Russian Far East, and Japan (Hokkaido). We developed the database of individual variants of B chromosome systems in *A. peninsulae*. In Siberian populations all animals had Bs. The karyotypes of the studied animals contain from 1 to 30 Вs differing in size and morphology. Analysis of B chromosome systems in 598 individuals from different localities of the range shows the presence of 286 variants of Вs combinations in these animals. Unique sets of B morphotypes make up most of these variants (64.7 ± 1.3%), probably suggesting that individual Bs systems normally result from stochastic processes in the populations. The proportion of animals with a large number of Bs gradually decreases, probably due to increased complexities in the inheritance of large numbers of Bs. *A. peninsulae* is thus proposed as a good model for studying the origin and evolution of extra elements in the karyotype.

## 1. Introduction

**Figure 1:**
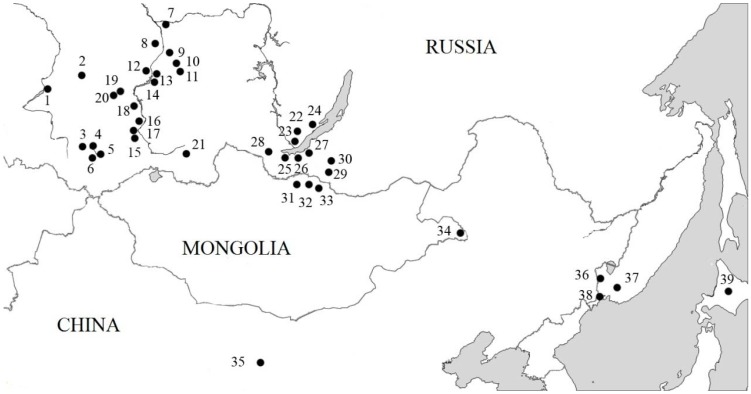
Geographical distribution of *A. peninsulae* and collection numbers. Arabic numbers beside points are location numbers shown in Table 1.

The Korean field mouse (*Apodemus peninsulae* Thomas, 1907) is widely distributed from East Siberia and North Mongolia, the Russian Far East to China, Korea, and Japan (Hokkaido) (Figure 1). *A. peninsulae* belongs to the genus *Apodemus*, in which six species have been shown to carry B chromosomes [1]. *A. peninsulae* shows one of the widest spectra of Bs variability among animals. Through the wide geographical range mice karyotypes contain from 48 to 78 chromosomes and the vast majority of individuals of this species have supernumerary (B) chromosomes [2,3,4,5,6,7,8,9,10,11,12,13,14,15,16,17]. The only population lacking B chromosomes is known from Sakhalin Island, Russia [10]. The Korean field mouse B chromosomes highly vary in morphology. Most mammals with Bs usually have B chromosomes of one type, such as acrocentric in *Apodemus (Sylvaemus) flavicollis* Melchior, 1834 or metacentric in *Rattus rattus* Linnaeus, 1758 [18]. In *A. peninsulae* up to five morphotypes were revealed [19].

Polymorphism of B chromosomes in *A. peninsulae* was found in the 1970s while investigating mice karyotypes from Hokkaido, Japan [20]. A range from small dot-like to large metacentric chromosomes was discovered. However, a relative homogeneity of Bs including only small and medium metacentric chromosomes was found in the mainland part of the species range (from Altai to Primorsky Region) [2,4,11]. Further expanding of catching localities in Central Siberia has demonstrated that mainland populations could also have a high variety of B chromosomes [12,14] leading to onward investigation of geographic variability of B morphotypes in the Korean field mouse [1,5,6,7,14,17,19,21,22,23,24,25,26].

The accumulated knowledge of morphological systems of B chromosomes in *A. peninsulae* raises new questions: how to estimate population variability of the species through patterns of its B chromosome variability; what meaning B chromosome morphology could have related to its molecular features; how B chromosomes are originated and inherited?

For over 40 years of the current study nearly 600 individuals of *A. peninsulae* from different parts of its range were karyotyped, making it possible to create an extensive database (http://sev-in.ru/ru/bdhromosomes-apodemus) and to analyze extra chromosome morphotypes in various geographically distant local populations. 

The purpose of the study was to identify common statistical patterns in the distribution of *A. peninsulae* B morphotypes in various populations of a wide range.

The Korean field mouse has thus become a good mammalian model for studies of evolutionary dynamics and effects of Bs on the host genome. The aim of this paper is to report new data on B chromosome distribution in local populations of the Korean field mouse *A. peninsulae* (Rodentia, Muridae) that would determine future directions for investigations.

## 2. Materials and Methods

We examined chromosomal data in 598 *A. peninsulae* individuals collected at 39 local populations in Russia, Mongolia, China, Korea, and Japan. 418 individuals from 30 localities were collected in Siberia (Central Siberia, Altai, Khakassia, Tyva, Baikal Lake region and Buryatia); 94 individuals were collected from Primorsky krai (the Russian Far East); 60 individuals were collected in Mongolia; 8 individuals were collected in Gansu province of China. Data on B chromosomes were partially published earlier [9,12,19,21]. The study protocol was approved by the Ethics Committee of the Severtsov Institute of Ecology and Evolution Russian Academy of Sciences (2017-03-17). All experiments with mice were performed in accordance with the rules approved by the European Convention for the Protection of Vertebrate Animals used for Experimental and other Scientific Purposes. We also used published data on 18 individuals from Hokkaido, Japan [20] (Figure 1, Table 1). 

Chromosome preparations were obtained from bone marrow and spleen cells after a routine technique with colchicine treatment [27]. At least 20 metaphase plates from each individual were taken for karyotype analysis. In this paper, we used only modal number of chromosomes; for mosaic animals (with one or none B chromosomes) we took those with one B. 

Our own developed formula was applied for morphotype numerical coding of B chromosomes [19]. The first number indicates the amount of Bs. The second number indicates B chromosomes of I class: large bi-armed chromosomes equal to 1–8 pairs of A chromosomes (large metacentrics). The third number indicated B chromosomes of II class: medium sized bi-armed chromosomes equal to 9–16 pairs of A chromosomes (medium metacentrics). The forth number indicates B chromosomes of III class: small bi-armed chromosomes equal to 17–23 pairs of A chromosomes (small metacentrics). The fifth number indicates B chromosomes of IV class: small acrocentric chromosomes equal to 17–23 pairs of A chromosomes. Finally, the sixth number indicates B chromosomes of V class or dot-like chromosomes (micro Bs). For example, the formula 5.1.1.1.1.1 means that an individual has five B chromosomes, one in each of five classes. In our opinion, both the ratio of different B morphotypes and the variety of total amount of chromatin in B chromosomes are highly significant to reveal the origin and specific existence of B chromosomes [22,23]. To estimate the amount of chromatin, we used conditional mass quantity of B chromosomes (mB index), developed by G.V. Roslik and I.V. Kartavtseva [13]. In this case, one conditional point is assigned to each size class: one point to dot-like (micro Bs) (V class), two points to larger and relatively similar in size chromosomes of IV and III classes, three points to II class and four points to I class chromosomes. The sum of points demonstrates a certain characteristic of Bs mass quantity.

Localities were aggregated according to distances and presence/absence of geographical, especially water, barriers between them.

The statistical analysis using standard techniques was conducted in Statistica 8.0 Software [28].

## 3. Results and Discussion

The analysis of 598 *A. peninsulae* karyotypes collected at 39 local populations in Russia, Mongolia, China, and Japan revealed presence of B chromosomes in 97.7% of the species population (Table 1). 12 individuals from the Russian Far East (12.8% of individuals collected in Primorsky krai) are the only exception with no B chromosomes in their karyotypes (localities no. 36–38, Figure 1). Table 1 summarizes the B chromosome data, including their number and morphology, in each examined locality. We also calculated the frequency of animals with macro and micro B chromosomes using our own information (Table 2).

**Figure 2:**
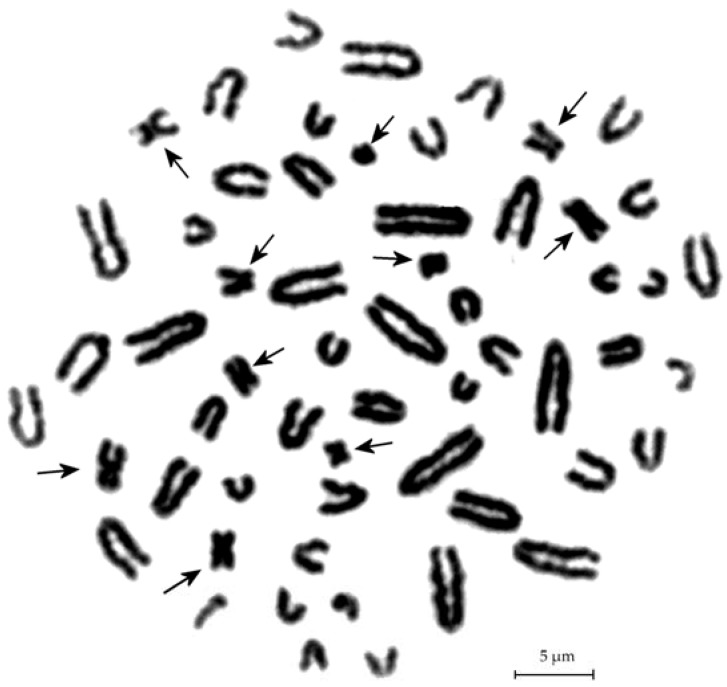
Metaphase plates of *A. peninsulae* with 10 different Bs.

**Figure 3:**
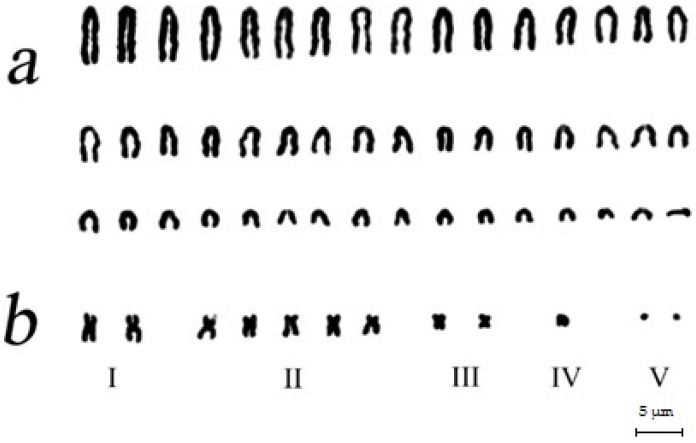
Karyotype of *A. peninsulae*: (**a**) the main set of 48 acrocentric A chromosomes and (**b**) variants of five (I–V) classes of B chromosomes. Formulas of Bs are 12.2.5.2.1.2.

The standard (A) diploid set of *A. peninsulae* contains 48 acrocentric chromosomes gradually decreasing in size (Figure 2 and Figure 3a). In addition, up to 30 B chromosomes may be found in some individuals. In this paper, we divide B chromosomes into two groups according to their size. The first group includes Bs of visible morphology under light microscopy (macro Bs), which are larger or equal in size to the smallest A chromosome. The second group includes only dot-like chromosomes which are much smaller than A chromosomes and without clear morphology (micro Bs). The macro B chromosomes are divided into classification types according to their morphology and relative size in comparison with the A chromosomes (Figure 3b). Despite high variability of B chromosomes, A chromosome aberrations were not found. 

We have previously described four types of macro Bs [12]: (1) large metacentrics or submetacentrics (Lm-sm); (2) medium-to-small metacentrics or submetacentrics (Mm-sm); (3) small metacentric (mm); (4) large-to-small acrocentrics or subtelocentrics (A-St). The most frequent macro Bs in *A. peninsulae* are Mm-sm (Figure 3a), whereas A-St morphological variants of medium or small size are rare. In some cases, we could identify the morphology of micro Bs in good quality metaphase plates, but in most cases micro Bs resembled very small structures without clear morphology. 

We suppose that dot-like (micro Bs) chromosomes are initial and originated by duplication of centromeric areas of A chromosomes with further reorganization into macro Вs through DNA amplification [22,23]. 

The frequencies of *A. peninsulae* individuals with one or another extra chromosome type are not equal in nature. Animals carrying even one medium or small metacentric B chromosome are more frequent (Table 2). Dot-like Bs also have relatively high frequency. In contrast, the frequency of large metacentrics across the Korean field mouse range is substantially lower; less than one third of mice caught in nature would have these chromosomes (*p* = 0.28 ± 0.018). Acrocentric B chromosomes, in their turn, are extremely rare. The similar tendency is shown for the Far East population of *A. peninsulae* based on the analysis of 367 individuals [13].

The number of large metacentric B chromosomes carried by a single karyotype varies from 0 to 5 across local populations. Their maximum number (4–5) occurs extremely rarely: only two such individuals were recorded in Siberia (Altai and Khakassia). Some mice from Altai, Novosibirsk and West Khentei (Mongolia) carried three large chromosomes, though one or two large B chromosome were met much more frequently. Meanwhile, the majority of examined individuals (72.2 ± 1.83%) did not have chromosomes of that type at all (Table 3).

**Figure 4:**
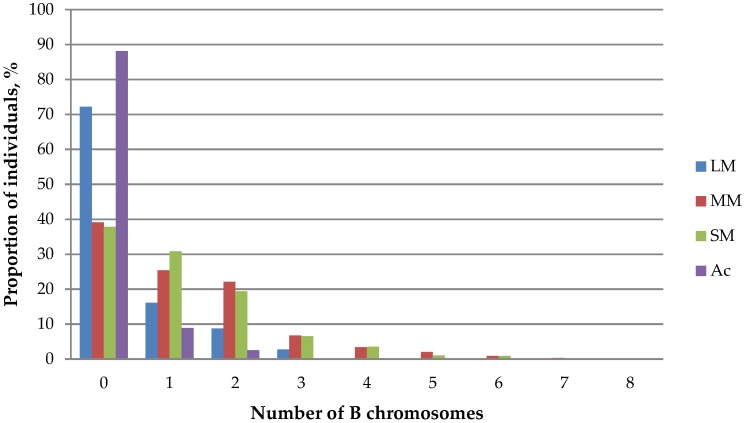
Ratio of *A. peninsulae* individuals with different numbers of B chromosomes across studied populations. LM: large metacentrics; MM: medium metacentric SM: small metacentric Ac: acrocentric.

The number of medium and small metacentric B chromosomes varies from 0 to 8 across different populations. Unlike macro metacentric chromosomes, their presence in populations is more significant as over 60% of karyotypes contain Bs of these types (Table 3). A tendency of decreasing proportion of individuals with increasing number of B chromosomes remains (Figure 4). The maximum number of medium (8) and small (8) metacentric chromosomes was recorded only once in karyotypes from left bank of Angara River and Novosibirsk, respectively. Mice with a small number of Bs (1–3) were recorded more often.

A tendency of distribution of acrocentric B chromosomes (IV class) in populations of *A. peninsulae* is similar with that of large metacentric chromosomes (Table 3, Figure 4). The maximum of acrocentric chromosomes (5) was found in a single karyotype from Novosibirsk area.

**Figure 5:**
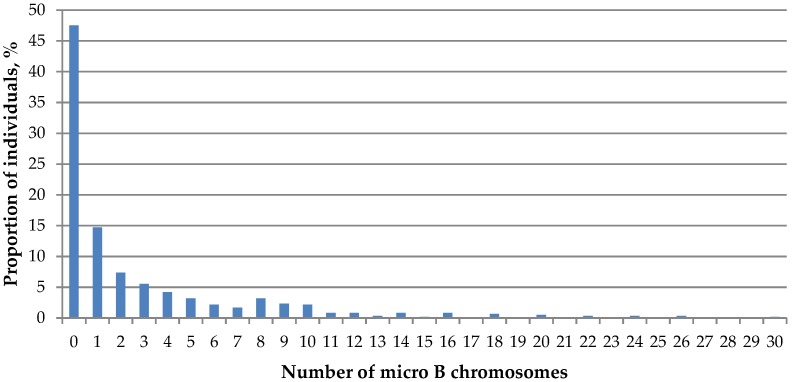
Ratio of *A. peninsulae* karyotypes with a certain number of micro B chromosomes across studied populations.

The number of extra dot-like chromosomes (micro Bs) in different populations varies from 0 to 30 (Table 3). Nearly half of examined Korean field mice (47.5 ± 2.04%) had no dot-like Bs. The proportion of mice with many dot-like Bs gradually decreased as in case of large B chromosomes (Figure 5).

It is necessary to emphasize that the correlation between the number and the frequency of chromosomes is rather high, negative, and significant in all cases (*p* < 0.05) (Table 4). This may indicate an increase in complexity of inheritance with an increase in the number of B chromosomes in the karyotype, particularly in case of large Bs (I, II and III classes).

**Figure 6:**
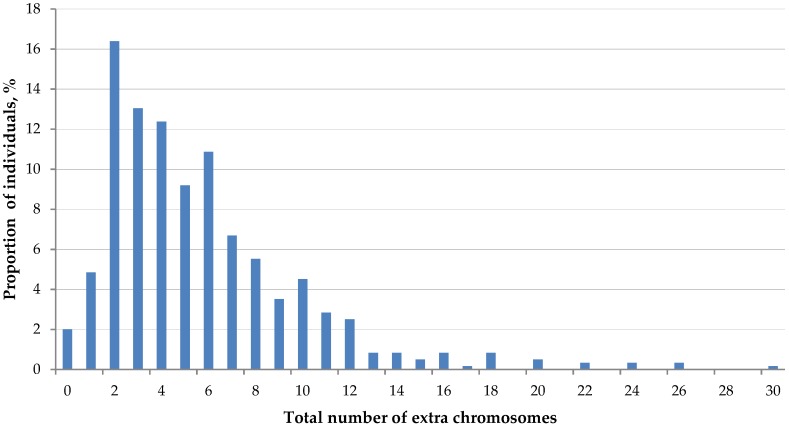
Ratio of *A. peninsulae* karyotypes with total number of extra chromosomes regardless of the type across examined local populations.

The frequency of *A. peninsulae* karyotypes with different numbers of all B chromosomes differs in some way from the frequencies of certain classes (Figure 6). Individuals with two B chromosomes are recorded much more frequently (16.39 ± 2.30%) than those with only one B chromosome type (4.85 ± 0.77%). In general, two thirds of mice in different local populations have from two to seven B chromosomes. Thus, we suppose that not just the presence of extra chromosomes, but the presence of several (not one) Bs in *A. peninsulae* karyotypes is normal for this species.

Overall, even and odd numbers of B chromosomes in a karyotype is not statistically equal (Pearson Chi-square test, χ^2^ = 6.64, *p* < 0.05). Individuals with an even number of Bs are recorded significantly more often (57.5%) that indirectly indicates the pattern of inheritance or chromosome division-merging processes.

The analysis of extra chromosome systems of 598 *A. peninsulae* individuals from different parts of the species range demonstrates the high variety of B combinations (286). Wherein, the unique Bs combinations are dominated (64.7 ± 1.3%, Table 5) that is likely due to the prevalence of stochastic processes in developing of individual Bs systems. Almost the third of examined animals (31%) carries the unique combinations of B chromosomes. 

**Figure 7:**
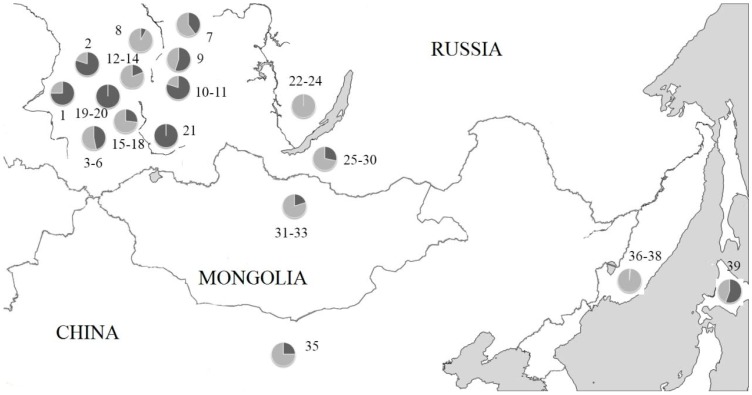
The proportion of animals in *A. peninsulae* local populations carrying unique B chromosome combinations (dark shading). Arabic numbers beside points are location numbers shown in Table 1.

The unique combinations of B chromosomes in mice karyotypes are met more frequently in local populations in Central Siberia and Japan (Figure 7). On the other hand, mice with repeated variants (non-unique) dominated in some well-studied populations such as local populations from the Far East (localities 36–38, *n* = 94), Western Baikal (localities 22–24, *n* = 28) and the left bank of Yenisei River (the locality 8, *n* = 26) (Figure 7).

Moreover, combinations with few numbers of Bs (0–4) are more frequent (the absence of B chromosomes was considered as one of 286 variants). The number of chromosomes in a combination negatively correlated with its frequency (Spearman’s rank test, r_s_ = −0.33, *p* < 0.05). In other words, the more there are B chromosomes in a karyotype, the less these combinations repeat across the species range and more often become unique. The most repeated was the combination of two small metacentrics recorded 31 times in different geographical regions: Khakassia, Western Baikal, Northern Mongolia and Primorye. The combination of one medium and one small metacentric was recorded 26 times and the combination of two medium metacentrics was recorded 15 times in Altai, Khakassia, Western Baikal, Northern Mongolia and Primorye populations. The variant with one small metacentric was found 22 times Khakassia, Western Baikal and Primorye populations. Combinations recorded twice constitute a considerable part (17.1 ± 0.7%).

The analysis of conditional mass quantity of B chromosomes (mB index) enabled us to compare samples with different amounts of extra chromatin in mice cells from different geographical populations. The lowest values were recorded in Primorsky krai (4.1 ± 0.3, Table 1) and the highest values were recorded in Kemerovo region and the south of Krasnoyarsk region (mB index > 18). Overall, the average mB index value across the western part of the *A. peninsulae* range (Central Siberia; 13.4 ± 0.35) almost twice higher than that across the eastern and southern parts (Baikal region, the Russian Far East, Japan, Mongolia, and China; 7.0 ± 0.26) (Student’s *t*-test, *t* = 13.94, *р* < 0.05). Meanwhile, neighboring local populations in Central Siberia could also substantially differ from each other by mB index. For instance, *A. peninsulae* populations from Krasnoyarsk region (localities 12–14, Figure 1) and the right bank of Yenisei River (locality 9) have relatively low mB index values (7.7 ± 0.5 and 11.2 ± 0.7, respectively, Table 1), although they are surrounded by the local populations with very high mB index values (14.5–18.7). A similar situation is found in Khakassia, where the mB index reaches 23.0 ± 4.6 (*n* = 3) in the north and 8.3 ± 0.7 in the south (Student’s *t*-test, *t* = 5.5, *р* < 0.05). 

**Figure 8:**
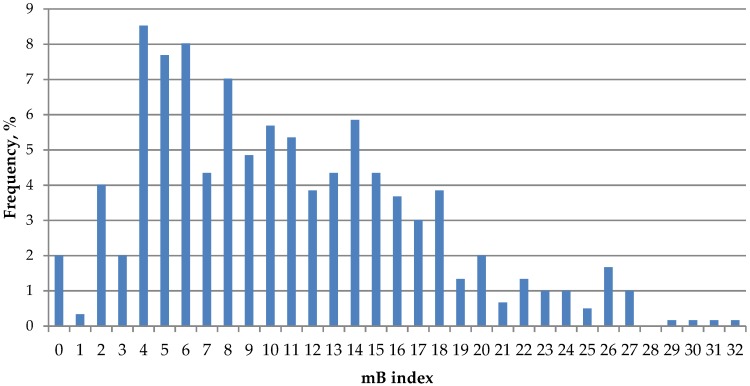
Frequency distribution of mB index values across studied *A. peninsulae* local populations.

The patterns of frequency distribution of mB index values (Figure 8) further reinforce the idea of adaptability of not just presence of B chromosomes but the presence of their certain mass in *A. peninsulae* cells. Mice carrying both very low and very high mB index value were rarely met in examined populations. More than half of individuals have mB index between 4 and 11.

In view of ecological prosperity of *A. peninsulae*, having wide species range and high adaptive potential, occupying various habitats, we assume that high Bs variety at least does not deteriorate the species adaptability. It is testified both by the fact that almost all karyotypes carry Bs and that Bs total masses within cells (mB index) have mainly average values. Many issues are likely to be removed after discovering Bs active genes or ascertaining an interaction system between A and B chromosomes. For instance, ribosomal genes of *A. peninsulae* have been already found [23].

From the other point of view, high polymorphism of quantity and composition of B chromosomes does not support the idea of strong control by the selection. The high proportion of unique B combinations evidently confirms it.

Thus, the specific features and the structure of B chromosomes, found in our research, make *A. peninsulae* a promising model for studying mammalian Bs. Further investigation of B chromosome population variability and its relation to other phenomenon of chromosome instability of DNA sequences in A and B chromosomes would enable the clarification of microevolutional processes in mammals.

## Figures and Tables

**Table 1:** B chromosome characteristics in the Korean field mouse *Apodemus peninsulae* Thomas, from different localities. For geographical map of localities see Figure 1. mB Index: mass quantity of B chromosomes.

No. of Locality	Locality	No. of Animals	No. of B Chromosomes			mB Index (M ± SE)
			Total	Macro	Micro	
Russia						
1	Novosibirsk Region	20	3–14	0–11	0–6	15.8 ± 1.4
2	Kemerovo Region	10	4–12	3–8	0–4	18.7 ± 2.1
3–6	The Altai Republic	146	1–10	1–10	0–3	14.5 ± 0.5
7	Angara mouth (between the left bank of the Angara River and the right bank of the Yenisei River)	15	6–9	6–9	0	18.5 ± 1.1
8	Central Siberia, the north of Krasnoyarsk territory, (the left bank of the Yenisei River)	26	4–30	0	4–30	18.1 ± 1.1
9	Central Siberia, the south of Krasnoyarsk territory, (the right bank of the Yenisei River)	40	3–18	0–6	1–14	11.2 ± 0.7
10–11	Central Siberia, the east of Krasnoyarsk territory, Zelenogorsk	5	8–17	3–4	5–13	17.6 ± 1.5
12–14	Central Siberia, Krasnoyarsk	47	1–10	0–4	0–8	7.7 ± 0.5
15–18	The south of Republic of Khakassia	30	1–10	1–6	0–7	8.3 ± 0.7
19–20	The north of Republic of Khakassia	3	5–9	5–9	0–1	23.0 ± 4.6
21	The Tyva Republic	2	12–15	2	10–13	15.5 ± 1.5
22–24	Western Baikal (Irkutsk Region, the western shore of the lake Baikal)	28	1–3	1–3	0	5.9 ± 0.4
25–30	Southern Baikal (The Republic of Buryatia)	46	2–13	0–5	0–12	11.3 ± 0.5
36–38	Primorye (Primorsky krai)	94	0–5	0–5	0–1	4.1 ± 0.3
Mongolia						
31–33	Northern Mongolia (West Khentei)	59	2–11	0–6	0–9	7.2 ± 0.4
34	The Great Khingan	1	1	1	0	3.0
China						
35	The Gansu Province	8	7–14	0–3	6–11	12.4 ± 1.5
Japan						
39	Island of Hokkaido	18	3–13	0–5	1–9	10.3 ± 0.9
	Total	598	0–30	0–11	0–30	10.7 ± 0.3

**Table 2:** Frequency of *A. peninsulae* individuals with different macro and micro B chromosomes in studied local populations.

Chromosome Type	Frequency of Individuals (P)	±SE
Large metacentric	0.28	0.018
Medium metacentric	0.61	0.020
Small metacentric	0.62	0.019
Acrocentric	0.12	0.013
Dot-like micro chromosome	0.53	0.020
No extra chromosomes	0.02	0.006

**Table 3:** Proportion of *A. peninsulae* individuals with different number of B chromosomes (%, ±SE) over the species range.

Number of B Chromosomes	Proportion of Individuals with B Chromosomes				
	Large Metacentric	Medium Metacentric	Small Metacentric	Acrocentric	Dot-Like (micro Bs)
0	72.2 ± 1.83	39.1 ± 2.00	37.8 ± 1.98	88.13 ± 1.32	47.5 ± 2.04
1	16.1 ± 1.50	25.4 ± 1.78	30.8 ± 1.90	8.9 ± 1.16	14.7 ± 1.45
2	8.7 ± 1.15	22.1 ± 1.70	19.4 ± 1.62	2.51 ± 0.64	7.36 ± 1.07
3	2.7 ± 0.70	6.7 ± 1.00	6.52 ± 1.00	0.17 ± 0.17	5.52 ± 0.93
4	0.17 ± 0.17	3.34 ± 0.74	3.5 ± 0.75	0.17 ± 0.17	4.18 ± 0.82
5	0.17 ± 0.17	2.0 ± 0.57	1.0 ± 0.41	0.17 ± 0.17	3.18 ± 0.72
6	-	0.84 ± 0.37	0.84 ± 0.37	-	2.17 ± 0.60
7	-	0.33 ± 0.23	-	-	1.67 ± 0.52
8	-	0.17 ± 0.17	0.17 ± 0.17	-	3.18 ± 0.72
9	-	-	-	-	2.34 ± 0.62
10	-	-	-	-	2.17 ± 0.60
11	-	-	-	-	0.84 ± 0.37
12	-	-	-	-	0.84 ± 0.37
13	-	-	-	-	0.33 ± 0.24
14	-	-	-	-	0.84 ± 0.37
15	-	-	-	-	0.17 ± 0.17
16	-	-	-	-	0.84 ± 0.37
18	-	-	-	-	0.67 ± 0.33
20	-	-	-	-	0.5 ± 0.29
22	-	-	-	-	0.33 ± 0.24
24	-	-	-	-	0.33 ± 0.24
26	-	-	-	-	0.33 ± 0.24
30	-	-	-	-	0.17 ± 0.17

**Table 4:** Correlation between the number and frequency of B chromosomes in karyotypes of *A. peninsulae* (Spearman’s rank test, *p* < 0.05).

	Type of B Chromosome				
	Large Metacentric	Medium Metacentric	Small Metacentric	Acrocentric	Dot-Like (Micro Bs)
r_s_	−0.986	−1.00	−0.98	−0.94	−0.9

**Table 5:** The number and ratio of B chromosome systems among *A. peninsulae* karyotypes across the species range.

Frequency of B Chromosome System across the Range	Number of Combinations	%	SE, %
Once (unique)	185	64.7	1.3
Twice	49	17.1	0.7
3 times	23	8.0	0.5
4 times	11	3.8	0.3
5 times	4	1.4	0.2
6 times	2	0.7	0.1
7 times	2	0.7	0.1
8 times	2	0.7	0.1
9 times	1	0.3	0.1
12 times	2	0.7	0.1
13 times	1	0.3	0.1
15 times	1	0.3	0.1
22 times	1	0.3	0.1
26 times	1	0.3	0.1
31 times	1	0.3	0.1
Total number of Bs systems	286		
